# Spectroscopic Study on *Pseudomonas Aeruginosa* Biofilm in the Presence of the Aptamer-DNA Scaffolded Silver Nanoclusters

**DOI:** 10.3390/molecules25163631

**Published:** 2020-08-10

**Authors:** Bidisha Sengupta, Prakash Adhikari, Esther Mallet, Ronald Havner, Prabhakar Pradhan

**Affiliations:** 1 Department of Chemistry and Biochemistry, Stephen F. Austin State University, Nacogdoches, TX 75962, USA; 2 Department of Physics and Astronomy, Mississippi State University, Mississippi State, MS 39762, USA; pa406@msstate.edu; 3 Department of Biology, Stephen F. Austin State University, Nacogdoches, TX 75962, USA; malletea@jacks.sfasu.edu (E.M.); havnerronal@sfasu.edu (R.H.)

**Keywords:** fluorescence, circular dichroism, partial wave spectroscopy, scattering

## Abstract

We report the effectiveness of silver nanocluster (Ag-NC) against the biofilm of *Pseudomonas aeruginosa* (PA). Two DNA aptamers specific for PA and part of their sequences were chosen as templates for growing the Ag-NC. While circular dichroism (CD) studies determined the presence of secondary structures, UV/Vis absorption, and fluorescence spectroscopic studies confirmed the formation of the fluorescent Ag-NC on the DNA templates. Furthermore, mesoscopic physics-based partial wave spectroscopy (PWS) was used to analyze the backscattered light signal that can detect the degree of nanoscale mass density/refractive index fluctuations to identify the biofilm formation, comparatively among the different aptamers with respect to the control sample. The importance of the secondary structure of the aptamer DNA in targeting, successfully binding with the cells and delivering the Ag-NC, is evidenced by the decrease in disorder strength (*L_d_*) of the Ag-NC treated samples compared to the untreated PA cells, which showed the abundance of higher *L_d_* in the PWS studies. The higher *L_d_* value attributed to the higher mass density fluctuations and the formation of biofilm. We envision this study to open a new avenue in using a powerful optical microscopic technique like PWS in detection, and DNA aptamer enclosed silver nanoclusters to prevent biofilms for opportunist pathogens like *Pseudomonas aeruginosa.*

## 1. Introduction

Bacterial biofilm formation is regulated by the cooperative activities and physiological processes of the microbial populations where cell to cell communication takes place by releasing small diffusible signal molecules which include secreted proteins, carbohydrates, and/or DNA [1,2]. *Pseudomonas aeruginosa* (Gram-negative bacteria) [3] forms biofilms by synthesizing three different polysaccharides—namely, alginate, Psl, and Pel—which help in the attachment of bacterial cells to the biotic or abiotic surface to make the highly structured multispecies communities [4,5,6,7] called biofilm. These exopolysaccharides (EPS) also protect multicellular aggregates from environmental stresses such as mechanical desiccation, pH shifts, osmotic shock, and UV radiation [6,7]. The phenotypes of the aggregated cells are distinct from those of planktonic cells.

The resistive properties of the biofilm to antimicrobial agents produce a major threat to public health. These threats include loss of host immune responses followed by chronic infections and even death in humans as well as causes major contaminations in the hospital, food, and environmental industries [8,9]. Studies on biofilms date back to 1877 by Ferdinand Cohn [10]. *Pseudomonas aeruginosa* virulent biofilms [11] cause critical infection in individuals with underlying health problems, which include cystic fibrosis pneumonia, chronic wound infections, chronic otitis media, chronic bacterial prostatitis, and contaminate medical device-related apparatus [12]. Several studies on biofilms of *Pseudomonas aeruginosa* have been carried out to understand different strategies of the emergence of biofilm and its control [2,4,5,7,8,13].

The ability to control the physicochemical properties of nanoscale materials has provided the means to use them in biomedical applications which resulted in the creation of a new field called “nanomedicine” [14]. Nanotechnology [14,15] has been demonstrated to reduce and control biofilm formation [14,16]. In recent years, some nanotechnology-based antimicrobials have been designed to kill antibiotic-resistant bacteria, but to combat biofilm-infections, additional noninvasive and non-toxic requirements must be met. Silver (Ag) is one of the most widely used metals in personal care, medical care, and household products [17]. Ag nanoparticles (NP) have been observed to prevent biofilm formation in Gram-positive and Gram-negative bacteria [18,19]. Our earlier work on *Bacillus thuringiensis* in 2016 [20] was the first report using silver nanoclusters (Ag-NC) as modes of prevention of the biofilm. Recently Wu, J. et al. have used Ag-NC on *Staphylococcus aureus* biofilm prevention [21]. However, both studies are on Gram-positive bacterial biofilm. In 2017 Soundy, J. et al.’s studies [22] on biofilm of *Pseudomonas aeruginosa* using DNA aptamers (small single stranded DNA/RNA stretches) specific for PA showed the aptamers can be the ideal candidates for modifications to be used as aptamer-drug conjugates and in biosensors. Aptamer conjugated gold nanoparticles were designed by Das, R. et al. in 2019 [23] to treat PA biofilms. The average size of silver nanoclusters falls in the range of 1–2 nm [24], compared to gold nanoparticles of 17 nm [23]. 

The objective of the present work is to exploit the PA targeted aptamers [25] as scaffolds in synthesizing functionalized Ag-NC in influencing the biofilm formation and study the mass density fluctuations of the cells in biofilm and planktonic states. Our objective is to obtain the nano-architectural changes of the PA biofilms in the presence of aptamers enclosed silver nanoclusters. Small sized nanomaterials can access the core of the biofilm better and impact its stability [20,25]. Two aptamer sequences were chosen following Das, R. et al. [23] and Soundy, J. et al. [22]. The nano-clustering properties of the nanomaterials were characterized by using optical spectroscopy which includes fluorescence and circular dichroism studies on the Ag-NC. The properties of biofilm formation/non-formation by the bacteria in the presence of Ag-NCs were characterized by using the partial wave spectroscopy (PWS) [26,27] on the PA biofilms with Ag-NC. The highly sensitive spectroscopic microscopy PWS technique allowed us to observe and characterize the biofilm in PA cells in absence of Ag-NC at the nanoscale, and study the prevention of the film formation directly in the presence of different Ag-NCs. This opened a new possibility for aptamer templated Ag-NC to act as a sensor for observing planktonic cells vs biofilm, as well as for its potencies in the prevention of biofilm formation.

## 2. Results

### 2.1. Characterization of the Aptamer-Templated Ag-NC

**Figure 1:**
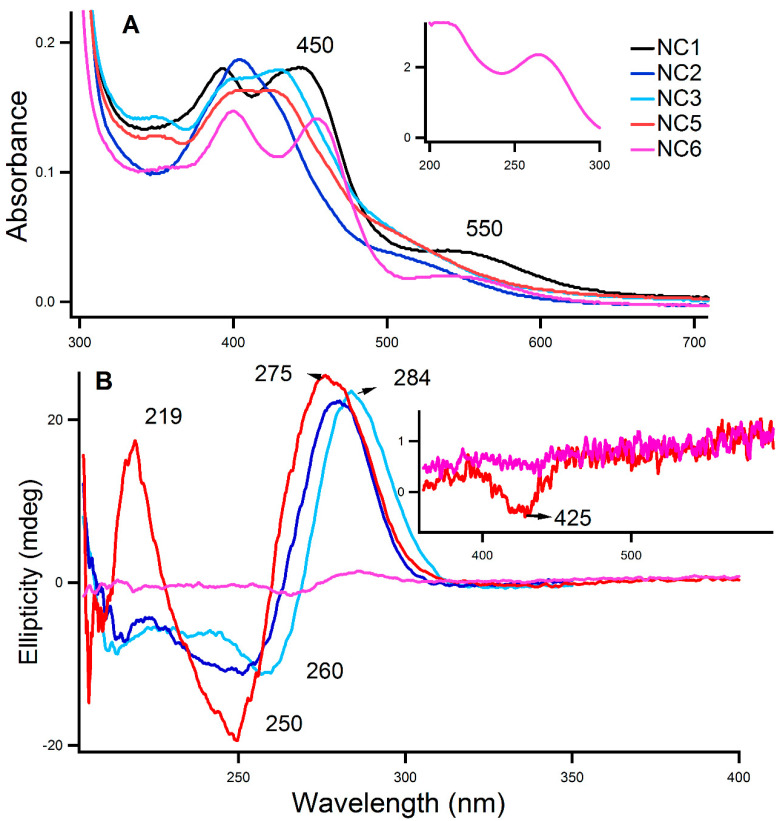
Absorption (**A**) and circular dichroism (**B**) spectra of aptamer-templated silver nanoclusters in water. The inset in 1A shows the absorption of the NC6 oligonucleotide in the UV region. 1B inset highlights the induced CD of NC5 in the visible region. [DNA] = 15 µM, [AgNO_3_] = 90 µM, and [NaBH_4_] = 90 µM. The sequences of the DNA in NC1, NC2, NC3, NC5, and NC6 samples are provided in the experimental section.

Figure 1A,B shows typical absorption and circular dichroism (CD) spectra of aptamer-DNA templated Ag-NC. The absorption of the nucleobases (in NC6) in the UV region is shown in Figure 1A inset which is similar to the other DNA sequences. Silver nanoparticles (NP) are formed during the process, as evidenced by the strong absorption band around 400 nm region [28]. The Ag-NCs are made in water with DNA (15 μM) and AgNO_3_ (90 μM), and reduced by NaBH_4_ (90 μM) to optimize nanoparticle formation [28]. A primary distinguishing feature of the nanoclusters is their strong absorption peak at wavelengths red-shifted that of 400 nm and narrower relative to the plasmon transition of the nanoparticles [28]. Examination of the absorption spectra in Figure 1A reveals a peak around the region of 460 nm with a shorter band at ~550 nm.

CD spectra provide diagnostic signatures for the presence of secondary structures in the single-stranded DNA sequence. The characteristic positive peak at ~285 nm and negative peak at ~260 nm indicate the formation of an i-motif structure, while a positive peak at ~265 nm and a negative peak at ~245 nm indicate the presence of B-DNA [29]. Our earlier study [30] indicated that Ag-NC influences the secondary structure of its scaffold DNA to some extent. Figure 1B presents the CD spectra of the Ag-NCs on the aptamer templates at room temperature in water. While the ellipticity profile of NC5 shows the presence of B-DNA (duplex) characteristics, the NC3 (the middle N_49_ sequence of NC5 aptamer) shows the formation of i-motif structure. The other aptamer sequence NC2 shows the presence of mixed secondary structures, indicating heterogeneity in the microenvironment of the Ag-NC on the aptamer-template. The presence of more guanine (G) than cytosine (C) in the left (NC6) primer (20 bases long) compared to the right primer (NC1), does not allow the NC6 sequence to form i-motif like structure as indicated in Figure 1B. Furthermore, Ag nanocluster induced CD was observed in NC5 in the visible region at 425 nm, as displayed in Figure 1B inset, which proves smaller nanoclusters retain the chirality of the DNA template which agrees to an earlier study [28].

**Figure 2:**
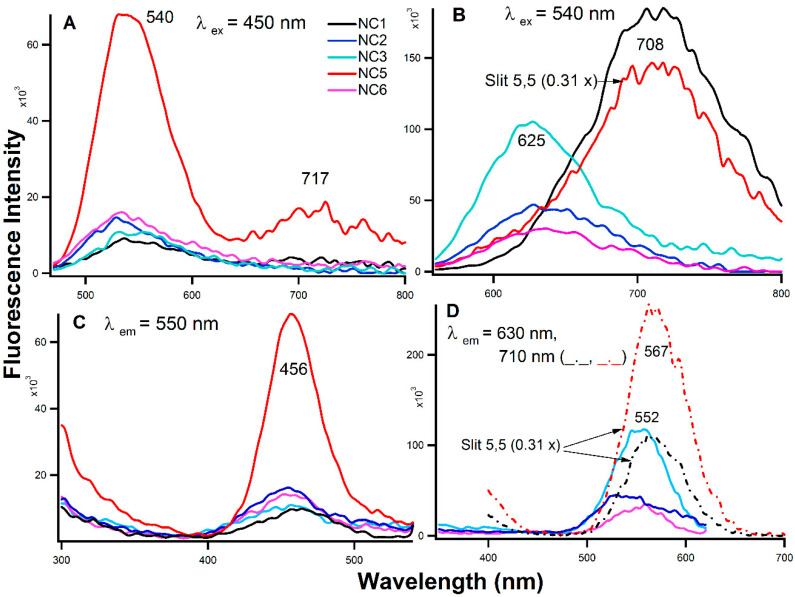
Fluorescence emission (**A**,**B**) and excitation (**C**,**D**) spectra of the aptamer-templated silver nanoclusters in water. [DNA] = 15 µM, [AgNO_3_] = 90 µM, and [NaBH_4_] = 90 µM. Excitation and emission slits were at 5 and 10 unless stated otherwise in the figure.

The absorption bands of the Ag-NCs around 450 nm and 550 nm in Figure 1A indicated the existence of more than one nanocluster on the templates. Figure 2A shows the emission profiles with $\lambda_{em}$ = 450 nm, where a strong emission band with $\lambda_{em}^{max}$ at 540 nm was observed for NC5 along with a second small band at ~717 nm. The NC on other templates showed less significant emission at 540 nm. With $\lambda_{em}$ = 40 nm (Figure 2B) however, NC1 showed the highest fluorescence at ~710 nm followed by NC5 whereas NC3, NC2, and NC6 developed the fluorescence emission band at 625 nm with gradual decreasing intensities respectively. The fluorescence excitation spectra in Figure 2C,D provide the wavelength of maximum excitation ($\lambda_{ex}^{max}$) at $\lambda_{em}$ = 550 nm (2C with $\lambda_{ex}^{max}$ at ~456 nm) and $\lambda_{em}$ = 630 nm (2D with $\lambda_{ex}^{max}$ at ~550/567 nm depending on the aptamer). This suggests that the sequence of the templates dictates the growth of the scaffolded Ag nanoclusters which agrees with our previous work [30,31,32]. It is pertinent to mention that the fluorescence excitation of NC2 and NC5 at $\lambda_{em}$ = 710 nm and emission of NC5 at $\lambda_{ex}$ = 540 nm were collected with slits (ex, em) 5, 5 due to the fluorescence intensity saturation with slits 5, 10.

### 2.2. Influence of the Aptamer-Enclosed Silver Nanoclusters on PA Biofilm

Incubation of the PA cells with different aptamer enclosed Ag-NC samples after 48 h gave rise to varying degrees of turbidity in the control and treated samples, which indicates the formation/non-formation of biofilm [20]. The highly sensitive optical microscopy technique, partial wave spectroscopy (PWS), as described above, can quantify any mass density or refractive index fluctuations/alterations in cells even at the nanoscale [33]. Here we used the PWS technique for the first time to probe the formation/non-formation of biofilms by measuring the degree of structural disorder or disorder strength. In particular, PWS identifies structural alterations in the PA cells forming the biofilm and can distinguish the effects of the different aptamer enclosed Ag-NCs in the formation of biofilm.

**Figure 3:**
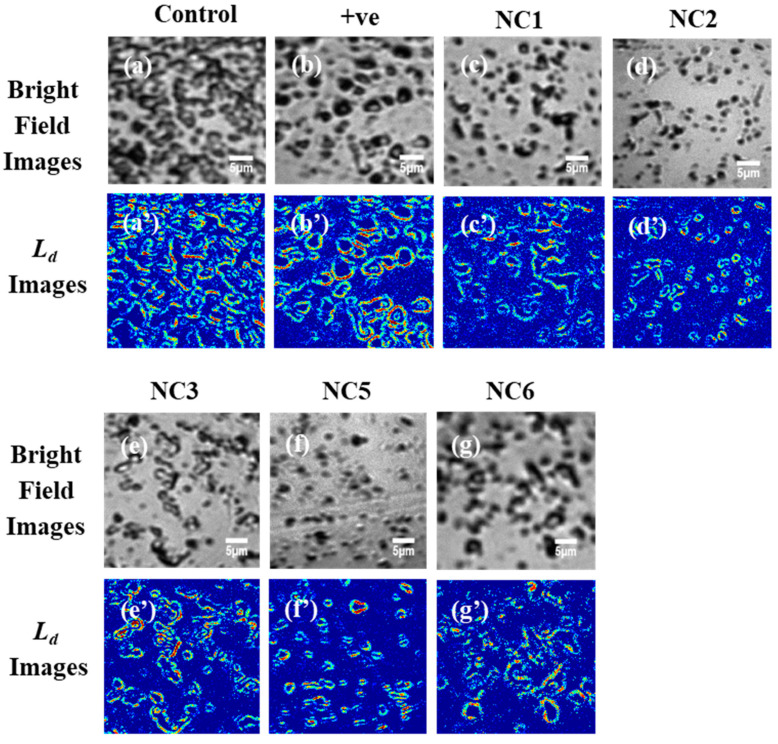
(**a**–**g**) are the representative bright field images of control, positive control (with AgNO_3_ + NaBH_4_) and different (NC1, NC2, NC3, NC5, NC6) aptamer enclosed nanocluster treated samples of *Pseudonomas aeruginosa* while (**a’**–**g’**) are their respective *L_d_* images. In the *L_d_* images, red spots represent a higher degree of refractive index or mass density fluctuations which are easily distinguishable. Long connecting bacterial structures are the sign of biofilm formation.

In Figure 3, the PWS analysis of the PA samples (control, positive control, and that with 25% of NC1, NC2, NC3, NC5, and NC6) are presented. As described in the method section, PWS experiments were performed and *R*(*x,y,λ*) data matrixes were obtained. From the data matrix, <*R*(*k*)>*_rms_* value and corresponding autocorrelation *C*(∆*k*) were obtained at every pixel point (*x*,*y*), which were used to calculate *L_d_* value using Equation (1). The degree of structural disorder or disorder strength of the mass density fluctuations or refractive index fluctuations at every point is represented in 2D/(*x*,*y*) of *L_d_* images (averaged over depth or z-direction of the film), is as shown in Figure 3.

Figure 3a–g display the bright field images of the PA control, positive control, and different Ag-NC treated cells, whereas the corresponding *L_d_* images are shown in Figure 3a’–g’. The red spots in the *L_d_* images (color maps) represent a higher disorder strength, i.e., the *L_d_* value at pixel (*x*,*y*) is higher. Compared to the bright field images, *L_d_* images are distinct since we can visualize the refractive index fluctuations in the sample. Here, the occurrence of more red spots in the *L_d_* image of the control PA compared to the Ag-NC treated ones indicated a pattern of a positive influence of the silver nanoclusters for preventing biofilm formation. The *L_d_* images made the distinction easier. The higher *L_d_* also related to the higher effectiveness of biofilm formation.

**Figure 4:**
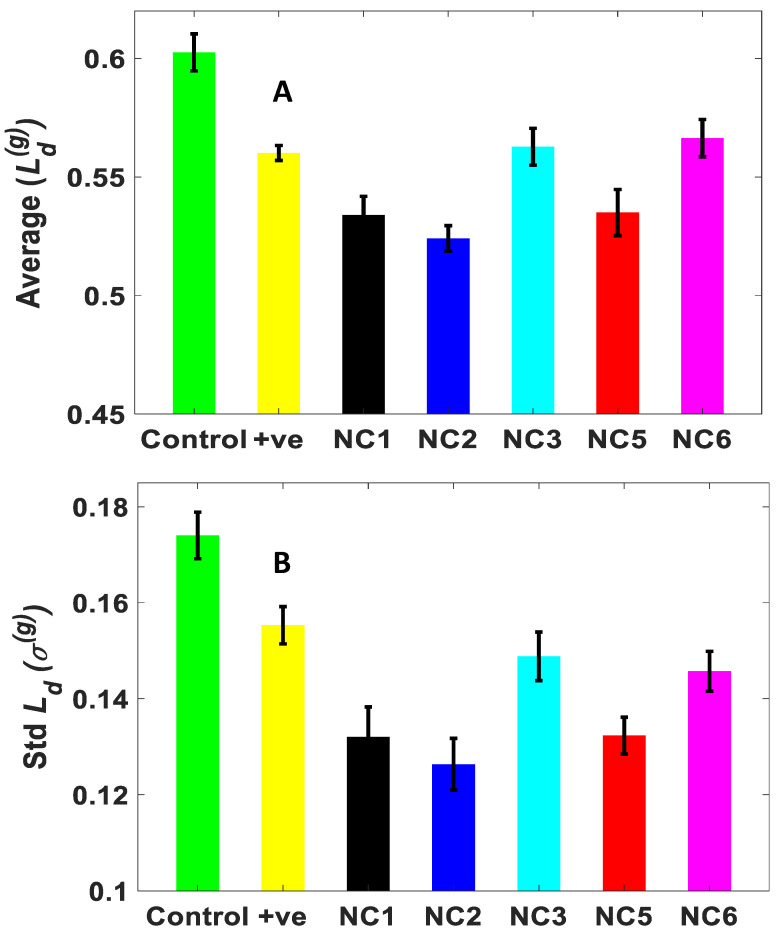
Average and standard deviation (*std*) of disorder strength *L_d_* measured by the PWS technique for PA bacteria film in the presence of different aptamer enclosed Ag-NCs. (**A**) The result shows that the average of the degree of disorder strength (*L_d_*^(*g*)^) of *Pseudonomas aeruginosa* cells decreases to a different extent in the presence of various nanocluster. (**B**) The result shows that the standard deviation of the degree of disorder strength (*σ*^(*g*)^) of *Pseudonomas aeruginosa* cells almost follow the same trend and decreases by 27% when treated with Ag-NC (*p* < 0.0001).

Figure 4A,B present the bar graphs of the average and standard deviation (*std*) of the disorder strength *L_d_* values with the standard error bars, respectively. The % differences in the average *L_d_* and its *std* (σ), between control, and (AgNO_3_ + NaBH_4_)/Ag-NC treated PA samples are displayed in Table 1. It can be seen from the bar graphs that the average (*L_d_*) and *std*(*L_d_*) have the same trend, this confirms the appropriateness of the use of the mesoscopic physics based PWS technique to the PA films on slides [26,27,33,34,35,36]. The decrease in the net *L_d_* wrt to the control followed the trend with NC2 > NC1 > NC5 > positive control ≈ NC3 > NC6 indicating the potency of an aptamer-DNA enclosed Ag-NC than just AgNO_3_ solution. It is shown that the disorder strength increases or decreases with the increase or decrease in the mass density fluctuations or refractive index fluctuations [26,27,34]. As can be seen in the *L_d_* images, the cells are moving apart, less overlapping, non-formation of the biofilm in Ag-NC treated PA, and decrease in the mass density fluctuations. Here the less decrease in the mass density fluctuations, *L_d_*^(*g*)^ in NC3 treated PA might be because NC3 sequence is a random cytosine rich sequence in the NC5 aptamer (the middle N_49_ sequence), the Ag-NC might not have interacted with the biofilm significantly. Likewise, PA samples treated with NC6 have rich guanine (G) NC6 sequence and has the least effect on the biofilm.

The average disorder strength, *L_d_*^(*g*)^ was found significantly higher in the PA control compared to treated cells (Student’s *t*-test, *p* < 0.0001). Similarly, the standard deviation (*std*) of the disorder strength *L_d_*, *σ*^(*g*)^ for the NC treated PA samples follows the almost same trend, pattern as the average disorder strength. However, as shown in Table 1 the percentage change *σ*^(*g*)^ in NC treated cells compared to the control has higher values than the mean *L_d_*^(*g*)^ (*p* < 0.0001).

## 3. Discussions

The hydrophobicity is an important aspect of creating the bacterial cell to cell adhesion [8]. The surface is considered hydrophobic, the bacterial clusters start clinging to the surface more than in solution due to less affinity for water than among themselves and the surface. Many studies [8,37,38,39] have been performed to understand the mechanism of bacterial adhesion to the surroundings (including surfaces and other cells) toward the biofilm formation. Apart from the hydrophobic attraction, electrostatic (due to the surface charge on the bacterial cell surface), and van der Waals attractive forces have also been proposed to be responsible for the initiation of biofilm [20,23]. Furthermore, surface thermodynamic analysis studies [40] added that the interfacial Gibbs free energies of adhesion play important roles in the formation of bacterial lawns on the surface. The EPS (exopolysaccharides) substances in PA not only protect the biofilm from external perturbations, but it also provides the matrix which contains water-filled channels for exchanging nutrients and excreting metabolic waste-products [16]. Due to size limitations, or by adsorption to the matrix, antimicrobial agents have difficulties in reaching the bacterial cell surfaces. Hence, it is imperative to use a delivery vehicle that can penetrate the matrix and deliver the drugs and kill the cell to cell adhesion. Single stranded DNA/RNA aptamers are of high prominence due to their relatively non-immunogenic and non-toxic properties, small size, and because they can be easily synthesized and modified making them an ideal candidate as drug transporters [22]. In this study, we have exploited this uniqueness of DNA aptamers in delivering 1–2 nm sized Ag-NCs (according to Petty, J. et al.’s studies in 2010, 2015) [32,41] to the EPS matrix, in order to identify the antibiofilm activities. We have used two different DNA aptamers sequences, a 60 bases long NC2 [23] and 89 bases long NC5 [22], along with parts of the NC5 sequence (NC1, NC3, NC6), which were used to grow the NCs. Through the CD spectroscopic measurements in Figure 1B, the secondary structures of the Ag-NC conjugated aptamer DNAs were characterized where NC5 shows the presence of the B-DNA structure. NC1 and NC2 displayed the presence of partial i-motifs, whereas NC3 (the C-rich middle stretch in NC5) showed strong i-motif forms. NC6 did not show a prominent secondary motif. The absorption and fluorescence studies as presented in Figure 1A and Figure 2 provide evidence of the formation of silver nanoclusters on the aptamer templates. Although NC5 aptamer showed the strongest emission followed by NC1 and NC3, all the sequences support the growth of metal nanoclusters to some extent.

The degree of structural disorder or disorder strength *L_d_*^(*g*)^ is significantly elevated in the control PA sample (high abundance of red spots in the *L_d_* image represent a higher disorder strength), providing the evidence of the formation of biofilm, as is depicted in Figure 3a’. The higher disorder strength is caused by higher mass density fluctuations due to the overlap of the PA cells and the formation of larger cell interfaces in biofilm. Compared to the control PA sample, all the nanocluster treated PA samples including that of positive control, show a decrease in the disorder strength to a varied extent. Table 1 provides the quantitative measures (%) of the decrease in the average and standard deviation (*std*) of *L_d_*. Between the two aptamers NC2 and NC5, the highest decrease in the average and *std* of *L_d_*^(*g*)^ values were observed for NC2. It is pertinent to mention that most of the DNA sequences we used to form stable secondary structures (as evident from CD profiles) which allowed them to bind to their target PA cell membrane with high affinity and specificity, potentially inducing a therapeutic effect, a theory which agrees with Soundy, J. et al. [25]. The shorter length of NC2 aptamer compared to NC5 might have contributed to its more potent nature to prevent the biofilm. The absence of a significant secondary structure in the left primer sequence of NC5 in water (NC6) contributed to its least potent nature as is seen in Table 1. The right primer (NC1) of NC5 has a significant i-motif structure which allowed the NC to get transferred to the PA cell membrane and prevent the cell from cell adhesion. The i-motif has a more compact structure than B-DNA according to our previous studies [29,31], which allowed the i-motif conjugated Ag-NCs to penetrate the EPS more. NC3 and positive control have a similar extent to decrease disorder strength. It should be noted that the DNA sequence in NC3 is a random cytosine rich sequence (to grow the metal nanocluster) and did not contain any part of an aptamer. Hence although NC3 had nanoclusters, the absence of aptamer did not allow the NCs to reach the cells. The NC6, NC3, and the positive control solutions contained silver nanoparticles and dissolved silver ions, which have attributed to the decrease in biofilm formation and disorder strength (*L_d_*^(*g*)^). This indicates a correlation between the effectiveness of biofilm formation and disorder strength parameter (*L_d_*^(*g*)^). It is noteworthy that silver nanoparticles have a larger size (10–100 nm) and exhibit some antimicrobial properties [42].

## 4. Materials and Methods

### 4.1. Choosing the Aptamer Sequences

Table 2 provides the DNA oligonucleotide sequences (with their abbreviations) which were chosen to synthesize the silver nanoclusters. The sequences comprised of two different aptamers NC5 [22] and NC2 [23], following Soundy et al. and Das, R. et al. respectively. The sequence NC5 consists of two specific primers underlined (left 5′-ATGAGAGCGTCGGTGTGGTA-3′ called NC6 and right 5′-TACTTCCGCACCCTC CTACA-3′ called NC1) and a middle 49 random bases long sequence (5′-CCC TTT CCC TTT CCC ATT CCC GTT CCC TTT CCC TTT CCC ATT CCCGTTA-3′ called NC3). The NC3 sequence is chosen as per our previous study [31,32] which is a cytosine rich sequence, expected to form Ag-NC. All these single stranded DNA sequences were custom-synthesized from Integrated DNA Technologies, Inc. (IDT, Coralville, IA, USA) and were chosen to make Ag-NC.

### 4.2. Preparation of DNA-Templated Silver Nanocluster

Triple distilled water (obtained from MilliporeSigma, St. Louis, MO, USA) was used to hydrate the lyophilized DNA oligonucleotides (IDT, USA) and make the Ag-NC solutions. The concentration of the stock DNAs was determined by absorbance using molar absorptivities based on the nearest-neighbor approximation. Silver clusters were synthesized by combining 15 µM DNA (the solution was kept in boiling water for 5 min to break all intramolecular interactions) and 90 µM AgNO_3_ solutions. Then an aqueous solution of NaBH_4_ was added to give a final concentration of 6 BH_4_- /oligonucleotide, and the resulting solution was vigorously shaken for 1 min. All samples were incubated overnight in the dark at 4 °C. UV/Vis absorption, fluorescence emission, and circular dichroism studies characterized the absorption, fluorescence emission, and chiral properties of the NCs.

### 4.3. Preparation of Bacterial Samples for Biofilm Study

*Pseudomonas aeruginosa* (PA) ATCC 10145, Lot 416-116-4 was supplied by Microbiologics Inc., MN. PA was grown in 200 mL Tryptic Soy Broth (TSB, FisherSci.) media at 23 °C for two days. Two six-well plates were used to incubate the bacterial culture with different Ag-NC solutions. 25% (*v*/*v*) of Ag-NC (NC1, NC2, NC3, NC5, NC6) solution was added to media containing a bacterial culture. PA in the presence of 100% media and 25%/75% (*v*/*v*) water/media (the same volume as for Ag-NC) did not show much difference in terms of bacterial growth in a separate study. Hence PA culture with 25% water in media is termed as a control in this work. The positive control is the sample which contained 25% aqueous solution containing 90 µM AgNO_3_ and 90 µM NaBH_4,_ which is known to form silver nanoparticles according to Mamun, R. et al.’s study [43]. The total volume in each well was 2 mL. The six-well plates (containing the PA control, PA positive control, and PA with the Ag-NC samples) were incubated at 23 °C for two days in six-well plates. Turbidity in the wells indicated the formation of biofilm, according to our previous work [20]. Cells from the plates were heat-fixed on the glass slides for the partial wave spectroscopic studies.

### 4.4. Techniques Used to Characterize the Ag-NC and PA Control/Treated with Ag-NC

#### 4.4.1. Steady State Absorption, Fluorescence, and Circular Dichroism

Steady state absorption, fluorescence, circular dichroism (CD) measurements were performed to characterize the silver nanoclusters formed on the DNA scaffolds. Steady state absorption spectra were recorded with a Shimadzu UV 2550 spectrophotometer. Steady state fluorescence measurements were carried out with a PerkinElmer FL 6500 fluorescence spectrophotometer. Excitation and emission slit widths were 5/10 nm. All reported luminescence spectra were corrected for the spectral response of the detector. Circular dichroism (CD) spectra were acquired with a J-810 spectropolarimeter (Jasco). The scan rate was 100 nm/min, and two consecutive spectra were averaged to produce the final spectrum. All CD measurements were performed at 25 °C in a 0.2 cm path length cell.

#### 4.4.2. Partial Wave Spectroscopy and the Quantification of Structural Disorder Strength (*L_d_*)

To understand the formation of the biofilm, we perform the structural disorder measurements of *Pseudonomas aeruginosa* bacteria films based on 1D backscattered light using the recently developed partial wave spectroscopy (PWS) [34] technique. Using the PWS method, we can detect the connectivity of the PA cells, as well as the biofilm formation, by measuring the degree of mass density/refractive index fluctuations, that is, the disorder strength parameter *L_d_.* The detailed experimental setup of an engineered finer focusing spectroscopic PWS system is presented elsewhere [26,27]. In the PWS technique, mesoscopic physics is combined with the spectroscopic imaging technique, to probe spatial mass density fluctuations or refractive index fluctuations in weakly disordered scattering medium such as cells. This finer focusing PWS system is highly sensitive enough to probe the nanoscale structural alterations due to abnormalities or changes in structural disorder [35,36]. Compared to conventional light scattering experiments, where a scattering signal is obtained by the interference of all waves propagating within the scattering particle in the far-field, the PWS technique analyzes the subset of the wave propagating in 1D in the backscattered spectrum. It is known that, in 1D scattering, the scattering signal brings maximum information from the sample. Therefore, due to the 1D probing nature of the PWS technique, sensitivity is enhanced to probe the refractive index fluctuations at the nanoscale level.

In PWS, scattering from a 3D disordered medium is expressed into a different parallel and spatially independent quasi-1D channels in *R*(*x,y,k*) reflection matrix, where *k* is the wave vector related to the probing wavelength *λ*, *k = *2*π/λ*. For each pixel (*x*,*y*), the variance of the refractive index fluctuation is computed as (Δ*n*^2^)^1/2^ of Δ*n(z).* The fluctuating component of the refractive index is collected using mesoscopic optical properties from the nanoarchitecture of the sample at any length scale below the diffraction limit. Hence, the degree of structural disorder strength, *L_d_*, at every pixel position (*x*,*y*) is derived from the *rms* value of the backscattered intensities <*R*(*k*)>*_rms_* and the spectral auto-correlation of the reflected intensities ratio, *C*(∆*k*) and is calculated as [26,27,33,34].

(1) $$L_{d}=\frac{Bn_{0}^{2}{\langle R\rangle }_{ms}}{2k^{2}}\frac{{\left(\Delta k\right)}^{2}}{-\ln\left(C\left(\Delta k\right)\right)}{|}_{\Delta k\to 0}$$

where B is the normalization constant, *n*_0_ is the average refractive index fluctuation of the weakly disorder media, and *k* is the wave vector with the probing wavelength λ (*k = *2*π/λ*).

For the Gaussian color noise, the degree of disorder strength can be further simplified as: *L_d_* = 〈Δ*n^*2*^*〉*l_c_*, which measures the physical state of the weakly disordered medium, where 〈Δ*n^*2*^*〉 and *l_c_* are the variance and the spatial correlation length of the refractive index fluctuations of the whole sample. At a given point in a cell, Δ*n* is proportional to the local mass density concentration of intracellular components and *l_c_* is the spatial correlation related to the size of the intracellular structures within a cell that are quantified using PWS. It has been shown that using the PWS technique, we can measure the change in the refractive index that happens as low as 20 nm [35] length scales. Thus, using the PWS, a 2D map that reflects the distribution of structural abnormalities at every point, i.e., *L_d_* (*x,y*) can be obtained (averaged along the depth z-direction for each (*x,y*) point) and are represented as *L_d_* images. Finally, the average and standard deviation (std) of disorder strength parameter *L_d_* for each sample are computed, then ensemble averaging was performed over a group of samples of the same category which are termed as *L_d_*^(*g*)^ and *σ*^(*g*)^ [35].

In the experiment, the collimated beam from a broadband stable white light source is used to probe a sample, and the backscattered spectral signals were recorded using the CCD camera for every wavelength in the visible range (450–700 nm) with the help of a liquid crystal tunable filter (LCTF). Hence, the recorded 3D data cubes are processed further as mentioned above to quantify the nanoscale structural abnormalities in terms of the statistical parameter, the mean, and standard deviation of *L_d_*.

## 5. Conclusions

In this work, we have developed a novel method to detect the biofilm of *Pseudonomas aeruginosa* by using the backscattered photons—a technique called PWS—which has come into high prominence in recent years. We have also proved that DNA aptamers can fruitfully be designed and exploited to make very small silver nanoclusters which can penetrate the exopolysaccharide matrix of the PA biofilm and prevent its formation. The proof of this principle was demonstrated by the decrease in the degree of mass density fluctuation or the degree of structural disorder strength (*L_d_*^(*g*)^) of the bacterial cells by using single stranded DNA aptamers targeted for PA as scaffolds to grow silver nanoclusters. The importance of secondary structures of the aptamer templates in delivering the Ag-NCs to the targeted cells is noteworthy. The increasing number of multi-drug resistant bacterial strains demand the intervention of new measures which are cost-effective, non-toxic, and easy to synthesize. This approach can be extended to other microbes which can strengthen the bridge between nanomaterial and microbiology research. Furthermore, the use of the recently developed highly sensitive and versatile partial wave spectroscopy (PWS) technique could accelerate the study of the structural properties of biofilms and other structural properties of bacteria at the nanoscale level, an area which needs more explorations.

## Figures and Tables

**Table 1:** Percentage difference in the average disorder strength (Δ*L_d_*^(*g*)^) and standard deviation (Δ*σ*^(*g*)^) between control *Pseudonomas aeruginosa* and treated samples.

Samples	Δ*L_d_*^(*g*)^ (%)	Δ*σ*^(*g*)^ (%)
Positive control	7.04068	10.7574
NC1	11.38513	24.1122
NC2	13.0183	27.3992
NC3	6.6087	14.4696
NC5	11.2092	23.9627
NC6	6.0012	16.2625

**Table 2:** Sequences of the single stranded DNA oligonucleotides used for synthesizing the silver nanoclusters (Ag-NC). The absorption wavelengths along with absorbance of the silver nanoclusters are provided in the third column. The left and right conserved primer sequences are underlined. The color designations are used to distinguish the sequences and are consistent in Figure 1, Figure 2 and Figure 4.

DNA Name	Sequence	Absorption Wavelength (nm) with Absorbance (OD) of the Ag-NC
**NC1**	**5′-TAC TTC CGC ACC CTC CTA CA-3′**	442 (0.18), 550 (sh)
**NC2**	**5′-CCC CCG TTG CTT TCG CTT TTC CTT TCG CTT TTG TTC GTT TCG TCC CTG CTT CCT TTC TTG-3′**	430 (0.15), 512 (sh)
**NC3**	**5′-CCC TTT CCC TTT CCC ATT CCC GTT CCC TTT CCC TTT CCC ATT CCC GTT A-3′**	431 (0.18), 511 (sh)
**NC5**	**5′-ATGAGAGCGTCGGTGTGGTA- CCC TTT CCC TTT CCC ATT CCC GTT CCC TTT CCC TTT CCC ATT CCC GTT A -TACTTCCGCACCCTCCTACA-3′**	427 (0.16), 511 (sh)
**NC6**	**5′-ATG AGA GCG TCG GTG TGG TA-3′**	454 (0.14), 560 (0.017)

## Abbreviations

*Pseudomonas aeruginosa*—PA; aptamer templated silver nanocluster—Ag-NC; nanocluster—NC; circular dichroism—CD; partial wave spectroscopy—PWS.
